# Prefrontal and striatal dopamine D_2_/D_3_ receptors correlate with fMRI BOLD activation during stopping

**DOI:** 10.1007/s11682-021-00491-y

**Published:** 2021-08-17

**Authors:** Philippe Pfeifer, Alexandra Sebastian, Hans Georg Buchholz, Christoph P. Kaller, Gerhard Gründer, Christoph Fehr, Mathias Schreckenberger, Oliver Tüscher

**Affiliations:** 1 University Hospital of Psychiatry and Psychotherapy, University of Bern, Bern, Switzerland; 2 Department of Psychiatry and Psychotherapy, University Medical Center of the Johannes Gutenberg University Mainz, Untere Zahlbacher Straße 8, 55131 Mainz, Germany; 3 Leibniz Institute for Resilience Research, Wallstraße 7, 55122 Mainz, Germany; 4 Department of Nuclear Medicine, University Medical Center of the Johannes Gutenberg University Mainz, Langenbeckstraße 1, 55131 Mainz, Germany; 5 Department of Neurology and Neuroscience, University Medical Centre Freiburg, Freiburg, Germany; 6 Freiburg Brain Imaging Centre, University Medical Centre Freiburg, Freiburg, Germany; 7 Brain Links-BrainTools Cluster of Excellence, University Medical Centre Freiburg, Freiburg, Germany; 8 Department of Molecular Neuroimaging, Medical Faculty Mannheim, Central Institute of Mental Health, University of Heidelberg, Mannheim, Germany; 9 Department for Psychiatry und Psychotherapy, Vitos Clinic for Psychiatry und Psychotherapy Hadamar/Weilmünster, Mönchberg 8, 65589 Hadamar, Germany

**Keywords:** Right inferior frontal gyrus, [^18^F]fallypride positron emission tomography, Striatum, OPRM1 gene, Stop-signal task

## Abstract

D_2_-like dopamine receptors in animals and humans have been shown to be linked to impulsive behaviors that are highly relevant for several psychiatric disorders. Here, we investigate the relationship between the fronto-striatal D_2_/D_3_ dopamine receptor availability and response inhibition in a selected population of healthy OPRM1 G-allele carriers. Twenty-two participants successively underwent blood-oxygen level dependent functional magnetic resonance imaging (fMRI) while performing a stop-signal task and a separate positron emission tomography (PET) scan. Striatal and extrastriatal D_2_/D_3_ dopamine receptor availability was measured using the radiotracer [^18^F]fallypride. Caudate D_2_/D_3_ dopamine receptor availability positively correlated with stopping-related fronto-striatal fMRI activation. In addition, right prefrontal D_2_/D_3_ dopamine receptor availability correlated positively with stopping-related striatal fMRI BOLD signal. Our study partially replicates previous findings on correlations between striatal D_2_/D_3_ dopamine receptor availability and response inhibition in a population selected for its genetic determination of dopamine response to alcohol and as a modulator of impulse control via the endogenous opioid system. We confirm the important role of D_2_/D_3_ dopamine receptor availability in the fronto-striatal neural circuit for response inhibition. Moreover, we extend previous findings suggesting that dopamine receptor availability in the right inferior frontal cortex, a crucial region of the stopping network, is also strongly associated with stopping-related striatal fMRI activity in healthy OPRM1 G-allele carriers.

**Supplementary Information:**

The online version contains supplementary material available at 10.1007/s11682-021-00491-y.

## Introduction

Impulsive behavior is of high clinical relevance in psychiatric disorders such as substance use disorders, attention-deficit/hyperactivity syndrome or personality disorders (Chamberlain & Sahakian, 2007; Robbins et al., 2012; Sebastian et al., 2012, 2013, 2014; Stahl et al., 2014; Turner et al., 2017). In the past years, response inhibition has come into focus of researchers as one component of impulsivity and has been established as a distinct and important neurocognitive function. Response inhibition is the ability to suppress a planned action or stop a repeatedly dysfunctional behavior (Bari & Robbins, 2013) and is often studied using the stop-signal task (SST) in animals as well as in humans (Logan & Cowan, 1984; Verbruggen & Logan, 2008). Applying functional magnetic resonance imaging (fMRI) studies, brain regions like right inferior frontal cortex (IFC) and anterior insula, pre-supplementary motor area (pre-SMA), striatum, and the subthalamic nucleus have been identified as being critically involved in implementing response inhibition (e.g., Aron, 2011; Chambers et al., 2009; Sebastian et al., 2016). Specifically, the fronto-basal-ganglia pathway has been implicated in stopping a response (Aron & Poldrack, 2006; Aron et al., 2014; Chen et al., 2020; Sebastian et al., 2013, 2016).

Addiction is one clinical field in which disturbed inhibitory control via fronto-striatal brain circuits plays a crucial role (Morein-Zamir & Robbins, 2015). Furthermore, there is evidence that both, impulsivity as well as addictive behaviors can be linked to a dysfunctional dopaminergic system (Bosker et al., 2017; Koob & Volkow, 2010). Pharmacogenetic studies provide links between fronto-striatal dopamine function and response inhibition (Colzato et al., 2009; Forbes et al., 2009). Neuroimaging studies revealed positive associations between striatal D_2_/D_3_ receptor availability and impulsivity in drug users (Buckholtz et al., 2010; Kohno et al., 2016). Dopaminergic neurotransmission has been shown to play an important role in the modulation of SST performance in rats (Bari et al., 2011). In humans, previous studies revealed associations between stop signal reaction time (SSRT), a measure of stopping latency, and dopamine release in frontal and precentral cortical regions as well as interactions of the striatal dopamine system and motor inhibition ability (Albrecht et al., 2014; Lorenz et al., 2015). In a behavioral study, SSRT was negatively correlated with D_1_- and D_2_-type dopamine Binding Potential (BP_ND_), specifically in the dorsal but not the ventral striatum. The effect of D_2_-type BP_ND_ on SSRT appeared to specifically relate to response inhibition while the relationship between D_1_-type BP_ND_ and SSRT seemed to represent a general motor effect (Robertson et al., 2015). To the best of our knowledge there is only one previous study in humans that used fMRI and Positron Emission Tomography (PET) to investigate the correlation between dopamine receptor availability and the neural networks of response inhibition. In line with Robertson et al. (2015), Ghahremani et al. (2012) reported that striatal dopamine D_2_/D_3_ receptor availability negatively correlated with stopping latency (i.e., SSRT). In addition, dopamine D_2_/D_3_ receptor availability positively correlated with inhibition-related fMRI activation in a fronto-striatal neural circuitry. Prefrontal clusters included ventrolateral prefrontal cortices and anterior insulae (Ghahremani et al., 2012), regions typically involved in response inhibition (e.g., Aron, 2011; Aron et al., 2014; Sebastian et al., 2013, 2016, 2017). Several lines of research thus provide ample evidence for a close link between fronto-striatal dopamine function and response inhibition.

In the present study we assess the relationship of fMRI BOLD signal during response inhibition and D_2_/D_3_ dopamine receptor availability in healthy male subjects. To this end, participants completed an SST during fMRI. D_2_/D_3_ dopamine receptor availability was measured using the radiotracer [^18^F]fallypride in a separate PET session. Findings on D_2_/D_3_ dopamine receptor availability in these subjects have been previously published elsewhere (Pfeifer et al., 2017). All participants of the study were originally selected for the A118G allele of the OPRM1 gene (rs1799971) with the aim to study alcohol effects in this genotype. Interestingly, preclinical and clinical studies also found that this genotype may modulate impulse control via the endogenous opioid system (Olmstead et al., 2009; Wiskerke et al., 2011; Ray & Hutchison, 2012).

In the light of the replicability crisis in the field of functional neuroimaging conducting replication studies is strongly required (Munafò et al., 2017). Therefore, using a similar study design as Ghahremani et al. (2012) the main aim of the present study was to replicate previous findings on D_2_-like dopamine receptors and their relationship to fronto-striatal brain activation in the context of impulse control. Given the findings by Ghahremani et al. (2012) and Robertson et al. (2015) we hypothesized to find a negative correlation of striatal D_2_/D_3_ receptor availability with stopping latency (i.e., SSRT). Moreover, we expected striatal D_2_/D_3_ receptor availability to positively correlate with fronto-striatal fMRI BOLD signal during response inhibition. In order to extend previous findings, we not only assessed the relationship of striatal D_2_/D_3_ receptor availability but also of prefrontal D_2_-like dopamine receptors with stopping-related brain activity.

## Methods

### Study design

For the present study all participants underwent three study visits: First, we assessed the medical history and conducted several basic medical tests (i.e., clinical examination, blood sample analysis, and drug urine screening test). On the second study visit, the participants underwent a structural MRI and performed an SST during fMRI. Third, D_2_/D_3_ dopamine receptor availability was measured by PET at a separate study visit.

### Ethical approval

Study procedures were in line with the Helsinki Declaration. Ethical approval was obtained by the local ethics committee of Rhineland Palatinate in Mainz, the radiation protection authorities (Bundesinstitut für Strahlenschutz—BfS) and the Federal Health Administration (Bundesinstitut für Arzneimittel und Medizinprodukte-BfArM).

### Participants

The participants for the present investigation were recruited as part of a larger study (Deutsche Forschungsgemeinschaft (DFG) – Project Number 126873260; https://gepris.dfg.de/gepris/projekt/126873260). Further study results have been reported in previous publications (Pfeifer et al., 2015, 2017). Participants were recruited via public advertisement. All recruited participants were genotyped for the *OPRM1* allele and only male 118G allele-carriers between 21 and 45 years of age who were non-smokers were included for data acquisition in this study. In total, 24 participants were included for the PET and fMRI measurements. Participants were financially compensated. Exclusion criteria were any actual or lifetime psychiatric and substance use disorder as well as current use of any psychotropic drugs. All participants were screened for psychiatric disorders with a standard psychiatric interview (“Diagnostic System for Experts” DIA-X; Wittchen & Pfister, 1997). Written informed consent was obtained from all participants prior to participation.

Two participants were excluded from all analyses (i.e., behavioral and neuroimaging) for not following task instructions resulting in a final sample of 22 participants. These participants had a mean age of 26.55 (SD = 4.62, range: 22–37) years. The IQ, based on the multiple-choice vocabulary test (MWT-B; Lehrl et al., 1995), was 105.50 (± 8.06, range: 96–129). On average, 8.31 days (SD = 1.15, range 3–21 days) elapsed between the fMRI and the subsequent PET measurement.

### Genotyping

The rs1799971 genotype was determined by pyrosequencing as described in a previous publication (Pfeifer et al., 2015).

### Stop-signal task

We employed the same experimental paradigm that was used in Sebastian et al. (2013). The task was programmed in Presentation software (version 13.0, www.neurobs.com). To ensure that the study participants understood the instructions of the task and to get familiarized with it, a brief training session was conducted prior to the scanning session. At the scanning session all participants accomplished three runs of the SST.

The SST consisted of a go condition (75%) and a stop condition (25%). A white fixation circle in the center of the screen was presented at the beginning of each trial (500 ms). Within this circle, a white arrow was presented for a maximum of 1000 ms or until a button press was performed. Participants were instructed to respond with a button press corresponding to the pointing direction of the arrows. In the stop condition, a stop-signal was presented after a variable stop-signal delay (SSD) following the display of the arrow. The stop-signal consisted of a color change of the circle from white to blue. Participants were instructed to attempt to stop the response in case of a stop-signal. The SSD was adapted to the participants' performance following a staircase procedure to yield a probability of 50% of correct inhibitions per run. In the beginning of a run, the SSD was 220 ms. If the response was not inhibited, the SSD was decreased by 50 ms in the next stop trial with a minimum SSD of 70 ms. If a response was inhibited correctly, the blue circle and the arrow remained on the screen and the SSD was increased by 50 ms in the next stop trial. The length of the interstimulus interval was jittered with a mean duration of 1000 ms and a standard deviation of 292 ms. A run consisted of 128 stimuli that were presented in a pseudo-randomized order (Sebastian et al., 2012, 2013).

### MRI data acquisition

Images were acquired on a Magnetom Trio syngo 3 T system (Siemens, Germany) equipped with a 12-channel head coil for signal reception. Stimuli were projected on a screen at the foot end of the scanner bore and were viewed with the aid of a mirror mounted on the head coil. Foam padding was used to limit head motion within the coil. A high-resolution T1-weighted anatomical data set was obtained using a 3D magnetization prepared rapid acquisition gradient echo (MPRAGE) sequence for registration purposes (TR = 1,900 ms, TE = 2.52 ms, flip angle = 9°, FOV = 256 mm, 176 sagittal slices, voxel size 1 × 1 × 1 mm^3^). Functional MRI images were obtained using T2*-weighted echo-planar imaging (EPI) sequence (TR = 2,200 ms, TE = 30 ms, flip angle = 90°, FOV = 192 mm, 36 slices, voxel size = 3 × 3 × 3 mm^3^).

### PET data acquisition

PET scans were acquired under resting conditions with closed eyes by means of a Siemens ECAT EXACT scanner (CTI, Knoxville, Tenn.) with an axial intrinsic resolution of 4.3 mm FWHM operating in the three-dimensional mode. Images were reconstructed by filtered back projection using a ramp filter and a Hamming filter (4 mm width). To correct for tissue attenuation, transmission scans were acquired with three rotating [^68^Ge]/[^68^ Ga] sources before injection of [^18^F]fallypride. Data acquisition comprised 39 time frames initiated immediately after the bolus intravenous injection of a mean of 171.4 ± 16.8 MBq of [^18^F]fallypride. The binning of the data increased progressively from 20 s to 10 min, resulting in a total scanning time of 180 min. The study participants remained in the PET scanner for the whole data acquisition time without a break (Pfeifer et al., 2017).

### PET Image analysis

The binding potential (BP_ND_) was calculated on a voxelwise basis using the simplified reference tissue model (SRTM) of Lammertsma and Hume (1996) (please see also Pfeifer et al., 2017). The cerebellum was chosen as a reference region since it is generally considered to be free of dopamine receptors. Prior to statistical analysis, the binding potential images were spatially normalized into Montreal Neurological Institute space (McGill University, Montreal) to remove intersubject anatomical variability. For this purpose, integral images (sum of frames between 4 and 8 min after infusion) were calculated and spatially normalized by using SPM8 routines (Wellcome Department of Cognitive Neurology, London) and a ligand-specific D_2_ template. Subsequently, transformation parameters of normalization were applied to respective individual BP_ND_ images (Pfeifer et al., 2017). For voxel-wise statistical analysis the spatially normalized BP_ND_ images were smoothed with an isotropic Gaussian filter of 12 mm at full width at half maximum (FWHM). The BP_ND_ was calculated on Volumes-of-Interests (VOI) by applying a VOI-template that we used in a preceding publication (Landvogt et al., 2010). Since BP_ND_ in the left and right regions of the given striatal VOIs (supplemental Fig. 1) were highly correlated (Caudate: *r* = 0.921, *p* < 0.001, 95% CI: 0.816–0.967; Putamen: *r* = 0.891, *p* < 0.001, 95% CI: 0.752–0.954) the average of the left and right portion of each VOI was used for correlation analyses. For the IFC we used the inferior frontal gyrus of the Hammers brain atlas (Heckemann et al., 2006). We chose to use the right IFC only since in particular the right IFC is implicated in stopping (Aron et al., 2014; Cieslik et al., 2015; Sebastian et al., 2013, 2016).

### fMRI image preprocessing

SPM 8 (http://www.fil.ion.ucl.ac.uk/spm/software/spm8), running with Matlab 8.2 (Mathworks Inc., Natick, MA) was used to conduct all image preprocessing and statistical analyses. Images were screened for motion artifacts prior to data analysis. The first five functional images of each run were discarded to allow for equilibrium effects. Several preprocessing steps were performed on the remaining functional images. First, images were realigned to the first image of the first run, using a six degrees-of-freedom rigid body transformation. The realigned functional images were co-registered to the individual anatomical T1 image using affine transformations. Subsequently, anatomical scans were segmented using the VBM8 toolbox (r435; http://dbm.neuro.uni-jena.de/software/). Deformation field parameters for non-linear normalization into the stereotactic Montreal Neurological Institute (MNI) space were derived from the DARTEL approach (Ashburner, 2007) implemented in VBM8 (using the provided MNI template of the IXI-550 cohort).

### fMRI single-subject analysis

A linear regression model (general linear model) was fitted to the fMRI data from all participants. Significant hemodynamic changes for each condition were assessed using *t*-statistics after convolution with a canonical hemodynamic response function. Five events were modeled: correct and incorrect reactions as well as omissions in the go condition and correct and incorrect reactions in the stop condition (successful and unsuccessful stop trials, respectively). Instruction and fixation cross were modeled as regressors of no interest. Head-motion parameters and their first derivatives as well as 1^st^ to 4^th^ order polynomial regressors of slow drift were entered as nuisance regressors. Prior to model estimation a standard 128 s high-pass filter was applied to the data and the model. As preprocessing and first-level analyses were conducted in individual space, resulting contrast images were transformed into stereotactic MNI space using DARTEL deformation fields and averaged across runs. Spatially normalized images were resampled to a resolution of 1.5 × 1.5 × 1.5 mm^3^ and smoothed with an isotropic Gaussian kernel with a full width at half maximum of 9 mm.

### fMRI group analysis

To assess stopping-related BOLD activity on second level, the parameter estimates resulting from the first level contrast 'successful stop > go' were entered into a second level, random effects group analysis using a one sample t-test design.

To assess correlations of stopping-related BOLD activity with BP_ND_, we used both voxelwise whole-brain as well as VOI-based approaches. On a whole-brain level, we subjected BP_ND_ within the right IFC, caudate and putamen to separate multiple regression analyses using the fMRI contrast 'successful stop > go'. All fMRI group results were thresholded at *p* < 0.05 corrected for multiple comparisons (family wise error, FWE, correction at cluster level using a height threshold of *p* < 0.001). The SPM anatomy toolbox 2.2b (Eickhoff et al., 2005, 2007) was used to allocate significant clusters of activation to anatomical regions.

For VOI-based correlations, following Buckholtz et al. (2010) we calculated the first eigenvariate of the parameter estimate for the contrast 'successful stop > go' for each subject for the following anatomically defined VOIs: the right inferior frontal gyrus (IFG) pars opercularis from the Harvard–Oxford atlas included in FSL (Desikan et al., 2006) and the left and right striatum VOIs from the probabilistic atlas from Keuken et al. (2014) (supplemental Fig. 1). All probabilistic masks were thresholded at 10%. We then used JASP (version 0.11.1; JASP team, 2018, https://jasp-stats.org) to compute Pearson correlations with D_2_/D_3_ receptor availability in right IFC, caudate and putamen. Based on previous findings (Ghahremani et al., 2012) we expected D_2_/D_3_ receptor availability to positively correlate with fronto-striatal fMRI data. We thus performed 1-sided tests for a positive correlation with a Bonferroni-corrected alpha threshold of *p* < 0.05/9 = 0.006.

### Behavioral data analyses

Behavioral data (RT and accuracy) were collected while participants performed the SST in the scanner. Measures of interest were mean RT on correct go trials, percentage of unsuccessful stop trials, and percentage of go omission errors. SSRT was computed using the integration method with replacement of go omissions with the maximum go RT following the recommendations of Verbruggen et al. (2019). Since previous studies reported a negative correlation of striatal BP_ND_ and stopping performance (i.e., SSRT) (Ghahremani et al., 2012; Robertson et al., 2015) we computed one-tailed Pearson correlations with a Bonferroni-corrected alpha threshold *p* < 0.05/3 = 0.017 for correlations with SSRT. To exploratory test for a relationship of fronto-striatal BP_ND_ and other behavioral measures of the SST (i.e., go RT and omission errors) we computed two-tailed Pearson correlations with a Bonferroni-corrected alpha threshold *p* < 0.05/6 = 0.008. For all behavioral data analyses, we used JASP (version 0.11.1; JASP team, 2018, https://jasp-stats.org).

## Results

### Behavioral results

Table 1 summarizes the behavioral results of the stop-signal task. The participants responded correctly on 98.56% of the go trials and successfully inhibited their response in 49.67% of the stop trials. Those results indicate that participants adhered to task instructions and that the staircase procedure worked successfully. SSRT and mean Go RT values corresponded to previous findings in the literature in comparable samples (Boehler et al., 2010; Ghahremani et al., 2012).

**Table 1:** Stop-signal task performance

	Mean	SD
RT correct go (ms)	441.23	85.82
RT unsuccessful stop (ms)	406.00	79.03
SSRT (ms)	199.77	53.11
SSD	224.09	110.28
% unsuccessful stop	50.69	3.46
% go omissions	0.68	1.11
% go choice errors	0.76	1.18

Percentage error trials (i.e., stop respond, go omission, go choice errors) was estimated by dividing the number of unsuccessful stop,go omission or choice errors on go trials by the total number of the respective trial type

*SSD* stop-signal delay, *SSRT* stop-signal reaction time

### Correlation of BP_ND_ and task performance

**Fig. 1:**
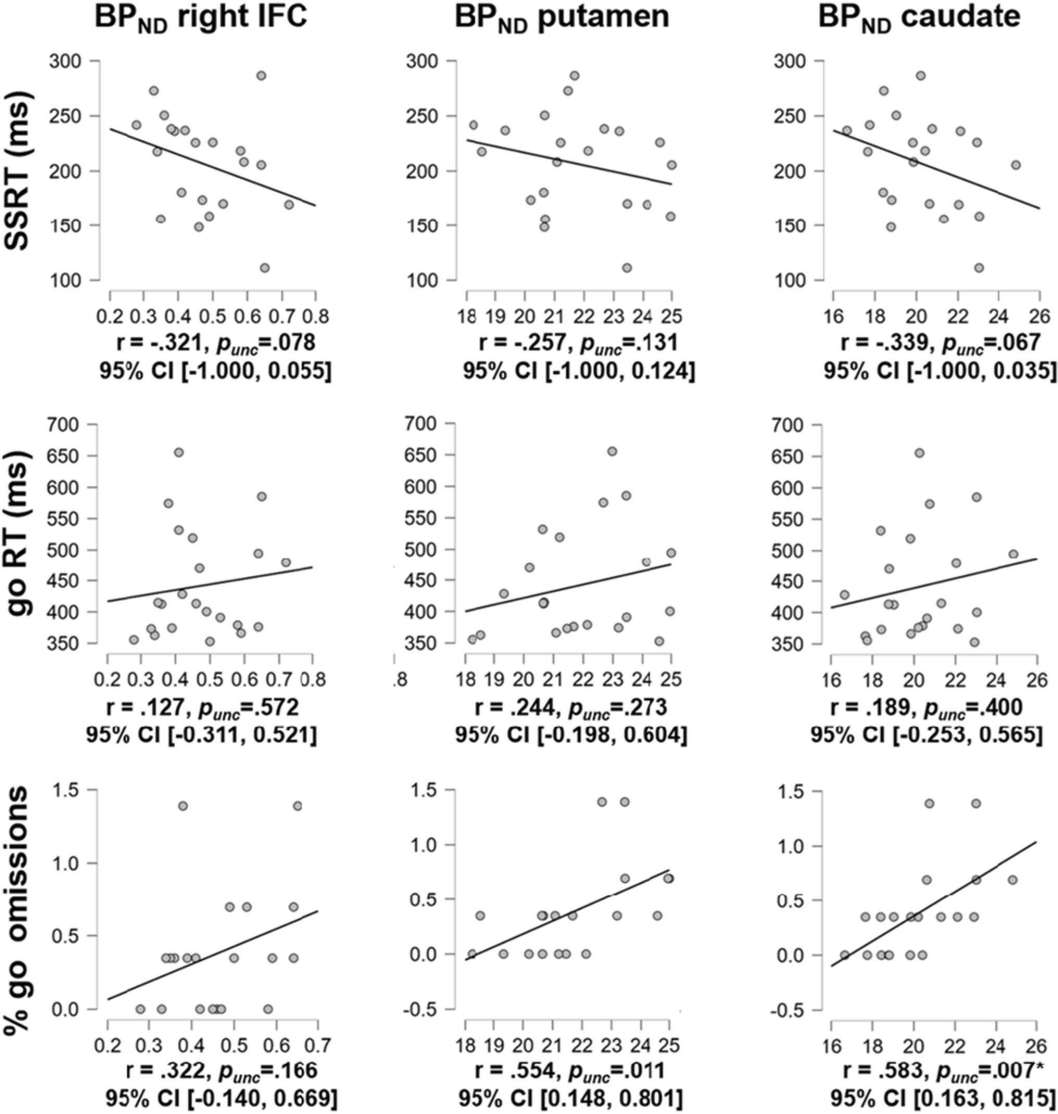
Correlations between binding potential (BP_ND_) in right inferior frontal cortex (IFC), putamen and caudate with performance in the stop-signal task (i.e., stop-signal reaction time (SSRT), go reaction time (RT) and % omission errors on go trials). * significant for Bonferroni-corrected alpha threshold *p* < 0.05/6 = .008

D_2_/D_3_ striatal receptor availability (BP_ND_) and its correlations with SST performance parameters are given in Fig. 1. D_2_/D_3_ striatal receptor availability in putamen, caudate and right IFC did not significantly correlate with stopping latency as indexed by the SSRT. Exploratory tests revealed a Bonferroni-corrected significant correlation of D_2_/D_3_ caudate receptor availability with go omission errors. We observed no further Bonferroni-corrected significant correlations.

### fMRI results

**Fig. 2:**
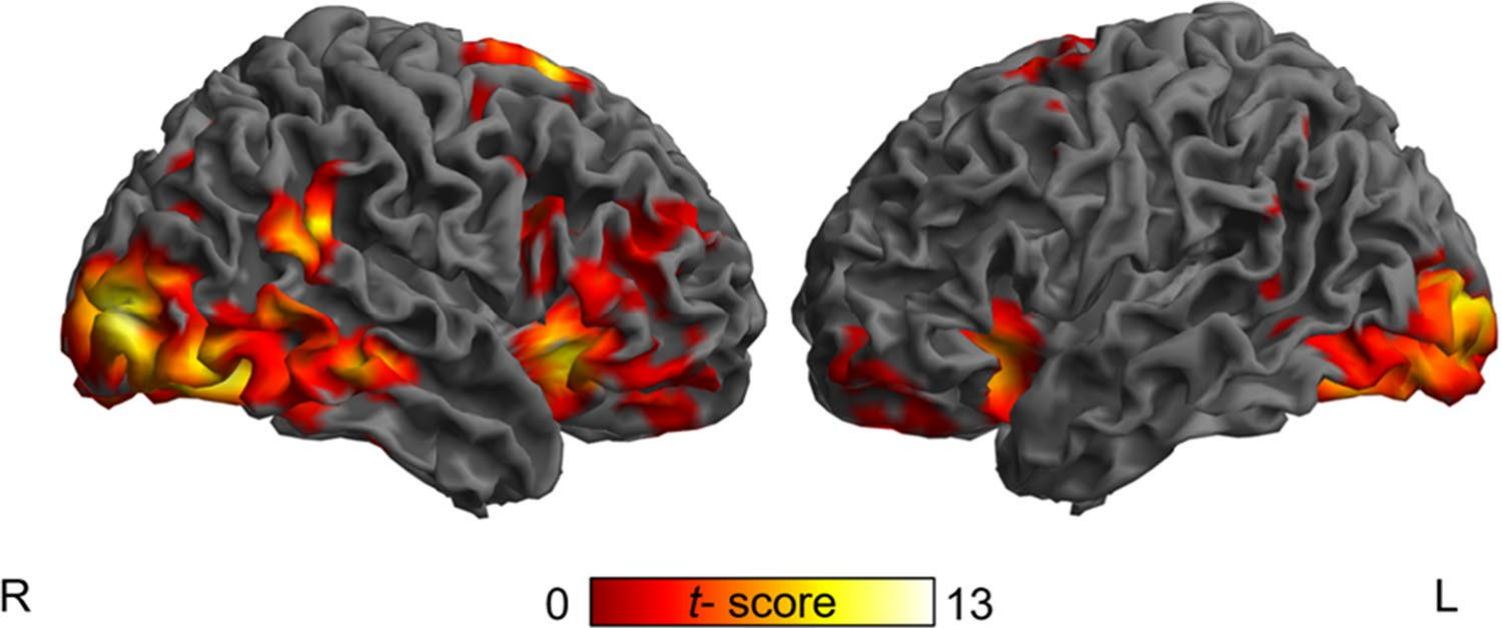
Activation maps for the contrast 'successful stop > go'. Maps are thresholded at *p*_FWE_ < 0.05, (correction at cluster level, cluster forming threshold of *p* < 0.001 uncorrected, min. cluster extent k = 1616 voxel). The color scale represents *t*-scores

Contrasting 'successful stop > go' resulted in activation typically associated with response inhibition in the SST. The activation pattern comprised significant clusters in a bilateral, but right lateralized fronto-parietal network. Prefrontal activation was mainly located in bilateral IFG/ anterior insula and pre-SMA (Fig. 2). Results are summarized in Table 2.

**Table 2:** Activation foci for the fMRI contrast 'successful stop > go'

Region	side	x	y	z	*Z*	*p*	k
Insula Lobe	R	36	17	-9	6.34	.001	13,696
IFG (p. Opercularis)	R	44	8	27			
Middle Orbital Gyrus	R	26	44	-18			
IFG (p. Triangularis)	R	48	29	9			
Middle Frontal Gyrus	R	42	36	18			
Olfactory cortex	R	17	12	-14			
Insula Lobe	L	-33	18	-9	5.96	.001	5573
IFG (p. Orbitalis)	L	-39	47	-17			
Middle Orbital Gyrus	L	-20	54	-18			
SuperioOrbital Gyrus	L	-17	36	-26			
Posterior-Medial Frontal Gyrus	R	12	17	65	6.16	.001	10,125
ACC	L	-2	27	30			
Superior Frontal Gyrus	R	18	5	71			
Posterior-Medial Frontal	L	-2	12	53			
MCC	R	11	18	41			
Superior parietal lobule	L	-62	-51	51	4.71	.002	1616
Middle Temporal Gyrus	L	-50	-53	-2			
SupraMarginal Gyrus	L	-56	-51	27			
Inferior Occipital Gyrus	R	36	-66	-12	6.71	.001	26,614
Middle Occipital Gyrus	R	33	-90	3			
Superior Temporal Gyrus	R	60	-44	23			
Fusiform Gyrus	R	35	-75	-14			
Calcarine Gyrus	R	20	-93	-5			
Middle Temporal Gyrus	R	51	-27	-11			
Fusiform Gyrus	L	-32	-62	-17	6.71	.001	13,232
Middle Occipital Gyrus	L	-21	-99	3			
Inferior Occipital Gyrus	L	-33	-87	-12			
Cerebellum (VI)	L	-38	-47	-29			
Cerebellum (Crus 1)	L	-26	-68	-30			

Local maxima of brain activations during 'successful stop > go' in Montreal Neurological Institute (MNI) x-, y-, and z-coordinates with associated Z-score (*p*_*FWE*_ < 0.05, cluster level corrected) and cluster extent in number of voxel (k). Coordinates of local sub-peaks within a cluster are shown indented

*ACC* anterior cingulate cortex, *MCC* middle cingulate cortex, *IFG* inferior frontal gyrus, *R* right, *L*  left

#### Voxel-wise analysis

**Fig. 3:**
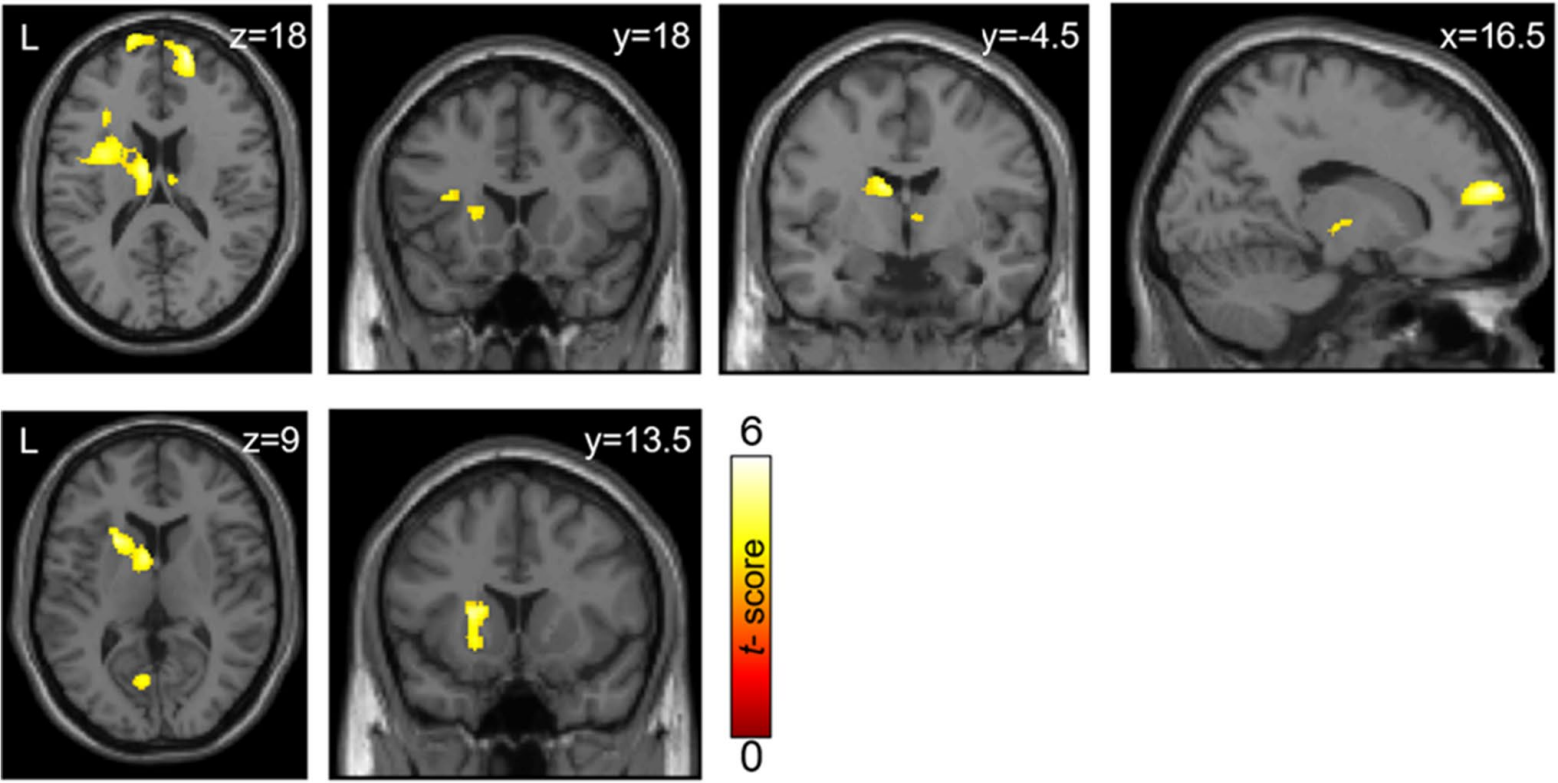
Activation maps for whole-brain voxel-wise multiple regression analysis using the fMRI contrast ' successful stop > go' and binding potential (BP_ND_) in the caudate (**A**) and right inferior frontal cortex (**B**) as covariates. BP_ND_ in the caudate correlated positively with brain activity in the left rolandic operculum, striatum and thalamus and with the superior frontal gyrus during successful stopping. D_2_/D_3_-receptor availability in the right inferior frontal cortex correlated positively with BOLD signal in the left striatum and calcarine gyrus during successful stopping. Maps are thresholded at *p*_FWE_ < 0.05, (correction at cluster level, cluster forming threshold of *p* < 0.001 uncorrected, min. cluster extent for a: k = 989 voxel and b: k = 772 voxel). The color scale represents *t*-scores

Whole-brain voxel-wise multiple regression analysis using the fMRI contrast 'successful stop > go' revealed a significant positive correlation of D_2_/D_3_-receptor availability in the caudate with one cluster covering the left rolandic operculum, caudate and thalamus and with the superior frontal gyrus (Fig. 3a; Table 3). D_2_/D_3_-receptor availability in the right inferior frontal cortex correlated positively with BOLD signal in the left striatum and calcarine gyrus (Fig. 3b; Table 3) during successful stopping. D_2_/D_3_-receptor availability in the putamen did not correlate with BOLD signal during successful stopping. There were no negative correlations between D_2_/D_3_ receptor availability in any region of interest with 'successful stop > go' BOLD signal.

**Table 3:** Activation foci resulting from whole-brain voxel-wise correlations of D_2_/D_3_-receptor availability (BP_ND_) with 'successful stop > go' fMRI activation

Region	side	x	y	z	*Z*	*p*	k
Caudate (mean) BP_ND_ correlation with 'successful stop > go' BOLD signal							
Rolandic Operculum	L	-36	6	14	4.50	< .001	3139
Caudate Nucleus	L	-9	-8	18			
Thalamus	R	3	-18	9			
Thalamus	R	14	-11	3			
Superior Frontal Gyrus	R	17	60	18	4.31	.018	989
Superior Frontal Gyrus	L	-15	68	15			
right inferior frontal cortex BP_ND_ correlation with ' successful stop > go' BOLD signal							
Putamen	L	-21	14	9	4.15	.011	1111
Caudate Nucleus	L	-6	5	9			
Calcarine Gyrus	L	-6	-71	12	3.88	.047	772
Lingual Gyrus	L	-9	-77	-2			

Local maxima of brain activations in Montreal Neurological Institute (MNI) x-, y-, and z-coordinates with associated Z-score (*p*_*FWE*_ < 0.05, cluster level corrected) and cluster extent in number of voxel (k). Coordinates of local sub-peaks within a cluster are shown indented

*BP*_*ND*_ binding potential, *R* right, *L* left

#### VOI-based correlations

**Fig. 4:**
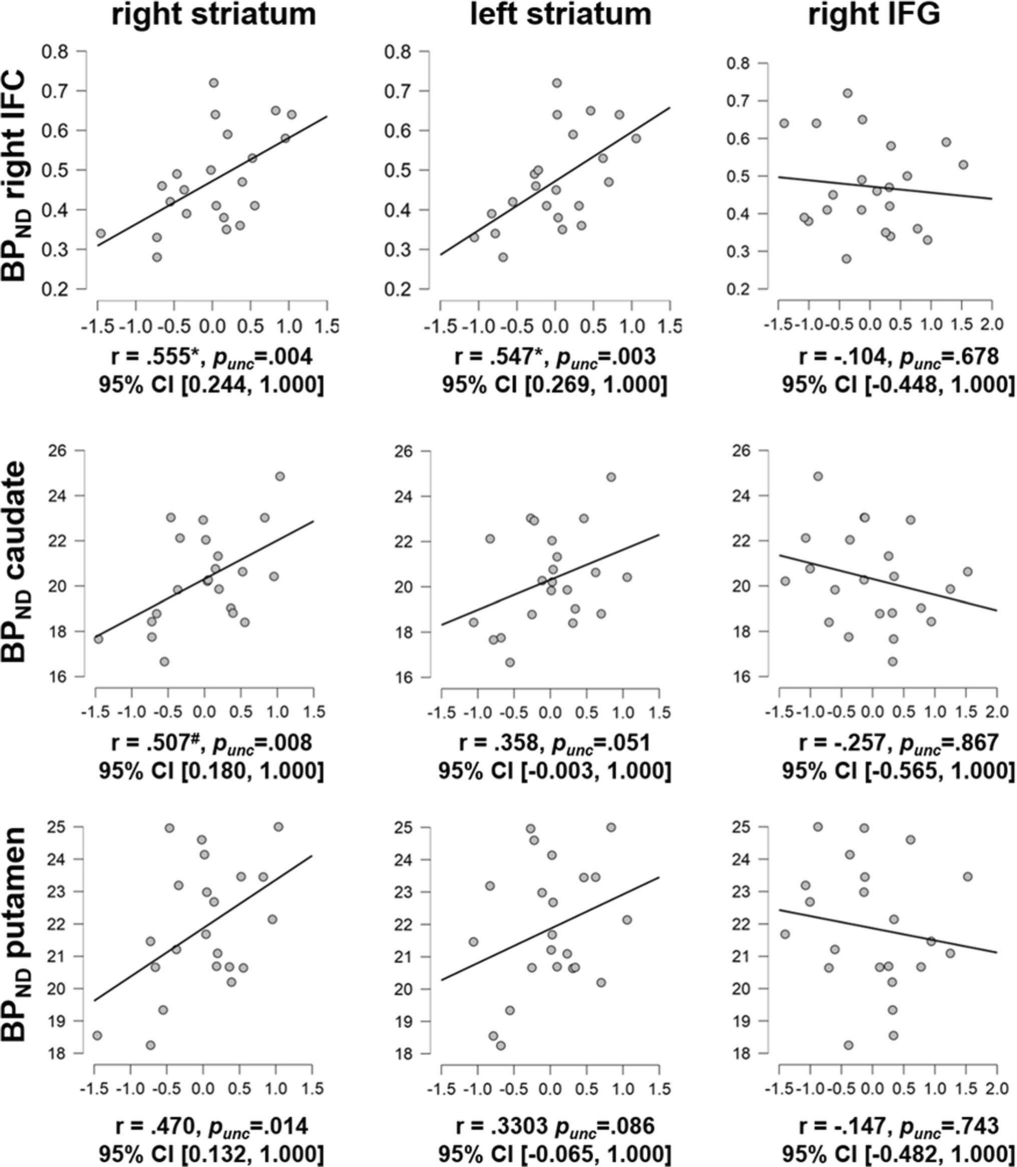
VOI-based correlations between binding potential (BP_ND_) in right inferior frontal cortex (IFC), caudate and putamen and BOLD signal in right and left striatum and right inferior frontal gyrus (IFG) during successful stopping (i.e., 'successful stop > go' fMRI contrast). * Correlation was significant after applying Bonferroni correction. ^#^ Correlation marginally significant after applying Bonferroni correction

After applying Bonferroni correction, we observed a marginally significant positive correlation of BP_ND_ in caudate with BOLD signal extracted from the right striatal VOI during successful stopping (i.e., with 'successful stop inhibit > go' fMRI activity). Furthermore, BP_ND_ in right IFC correlated positively with bilateral striatal BOLD signal during successful stopping (Fig. 4). For none of the VOIs a Bonferroni-corrected significant correlation of BP_ND_ with right IFG fMRI BOLD signal was observed.

##### Discussion

In the present study we sought to replicate previous findings on the relationship of D_2_/D_3_ receptor availability and stopping latency and associated fronto-striatal brain activation in the context of impulse control in a sample of healthy OPRM1 G-allele carriers. In contrast to previous findings (Ghahremani et al., 2012; Robertson et al., 2015) we did not observe a significant negative relationship of stopping latency (i.e., SSRT) and fronto-striatal D_2_/D_3_ receptor availability. Yet, fronto-striatal D_2_/D_3_ dopamine receptor availability significantly correlated positively with BOLD activity during successful stopping as expected. More specifically, whole brain voxel-wise multiple regression analyses revealed that D_2_/D_3_ receptor availability in the caudate was significantly associated with stopping-related prefrontal and caudate fMRI activity. In addition, we assessed the relationship of D_2_/D_3_ receptor availability in the right IFC, since the right IFC has been shown to be crucial for stopping (Aron et al., 2014; Sebastian et al., 2016, 2017). D_2_/D_3_ receptor availability in the right IFC was significantly associated with striatal BOLD signal during successful stopping. VOI-based analyses largely confirmed the whole-brain results. We observed a highly significant positive correlation of fMRI parameters extracted from bilateral striatal VOIs with right IFC BP_ND_ as well as a marginally significant correlation of fMRI parameters extracted from the right striatum with right caudate BP_ND_. Taken together, the present findings provide further evidence for the important role of striatal D_2_/D_3_ receptor availability in humans for the neural circuitry subserving response inhibition behavioral control. Moreover, we show that also right prefrontal D_2_/D_3_ receptor availability is critically related to fronto-striatal brain activity during successful stopping.

### D_2_/D_3_ -receptor availability and response inhibition

The modulatory role of dopamine in inhibitory action control is broadly supported by pharmacological testings and observations in various clinical populations (de Wit et al., 2002; Colzato et al., 2009; Lee et al., 2016, Nandam et al., 2013). A previous study demonstrated that disturbances within ascending dopaminergic trajectories (i.e., decreased midbrain D_2_/D_3_- autoreceptor availability) may enhance deficits in impulse control as assessed with the Barratt Impulsiveness Scale (Buckholtz et al., 2010). In contrast to the behavioral paradigm used in the present study impulsivity was measured as a personality trait in the study by Buckholtz et al (2010). This is important to note since associations between impulsive traits measured with self-report scales and state impulsivity as assessed using experimental paradigms are – if at all present – relatively small (Cyders & Coskunpinar, 2011; Reynolds et al., 2006; Stahl et al., 2014). Results are thus not directly comparable. Here, we applied a neurocognitive impulse control task with the aim to replicate findings from preceding studies that investigated the contribution of striatal dopaminergic function to inhibitory control in humans with a similar methodology (Ghahremani et al., 2012; Robertson et al., 2015). In contrast to previous findings, SSRT displayed only a non-significant negative association with D_2_-like dopamine receptors in the present study. Therefore, the data do not support strong associations between D_2_- like dopamine receptors in the dorsal striatum and inhibitory control. It is noteworthy, however, that brain behavior correlations in small samples (*n* = 20–30) may be sensitive to outliers and may overestimate the actual effect size (Cremers et al., 2017). Brain-behavior correlations in the present and previous studies were assessed in small samples (present study: *n* = 22, Ghahremani et al., 2012: *n* = 18, Robertson et al., 2015: *n* = 27). Nevertheless, the association of SSRT and striatal D_2_/D_3_-receptor availability was consistently negative in all of these studies providing accumulating evidence for the suggested relationship. Ghahremani et al. (2012) as well as other authors point out that models of behavioral control should consider not only D_2_-like receptor availability, but also their balance with the D_1_ receptor status in the striatum (Eagle et al., 2011). As the latter cannot be measured with [18F]fallypride, our study cannot provide information on the ratio of both different receptor types. This may, however, be essential for an integrative interpretation of D_2_-like receptor availability and its correlation with stopping latency.

One interesting finding in our study population is that elevated D_2_/D_3_ dopamine receptor availability in the caudate was associated with a higher propensity for go omission errors. In line with that, in a study that investigated the neural correlates of omission errors using event-related potentials omissions were attentional lapse-based errors as indicated by a delayed brain preparation before the stimulus onset (Perri et al., 2017). Furthermore, significantly elevated omission error rates in the SST have been reported in individuals with ADHD, a clinical condition that may benefit from a stabilization of dopamine signaling when treated with stimulants (Adams et al., 2011; Lee et al., 2016; Overtoom et al., 2002).

### Stopping-related BOLD activity and striatal D_2_/D_3_-receptor availability

Multiple regression of caudate D_2_/D_3_ receptor availability revealed a positive association with stopping-related fMRI activity in the rolandic operculum, caudate, thalamus and superior frontal gyrus, thus replicating previous findings by Ghahremani et al. (2012) in large parts. The present results extend previous findings by additionally testing for stopping-related associations with right prefrontal D_2_/D_3_-dopamine receptor availability. Right IFC BP_ND_ was associated with left striatal activity resulting from the 'successful stop > go' fMRI contrast. This finding highlights the possible relationship of prefrontal dopamine transmission for stopping and strengthens previous findings of a lateralization of this brain function (Aron et al., 2014). However, our data cannot reveal causal relationships between dopamine transmission and inhibitory control. Therefore, the question remains how dopamine exactly modulates the stopping network. In this context, Kayser et al. (2016) assessed the changes in impulsive choice ratio on tolcapone, a drug that augments frontal dopamine tone, in a cohort of pathological gamblers using a randomized, double-blind, placebo-controlled within-subject study design. Stronger baseline right IFC BOLD signal correlated with greater declines in impulsive choice on tolcapone versus placebo. In addition, connectivity of right IFC and striatum increased on tolcapone versus placebo. This suggests that tolcapone or dopamine augmentation in general may have a role in reducing impulsive behaviors, specifically via increases in top-down control via fronto-striatal pathways in particular in individuals with greater baseline right IFC activity. The findings by Kayser et al. (2016) thus underline the present findings regarding the importance of fronto-striatal dopaminergic modulation in the context of impulse control. Furthermore, researchers who investigated cortical dopamine release during an SST with fallypride PET found that changes in cortical D_2_/D_3_ receptor availability were detectable (Albrecht et al., 2014). Interestingly, the latter study demonstrated significant changes in cortical dopamine in anatomic locations corresponding to neural correlates of inhibiting motor responses as characterized in humans with fMRI (left orbitofrontal cortex, right middle frontal gyrus, and right precentral gyrus).

Taken together, the present results corroborate a positive association of stopping-related striatal brain activity with D_2_/D_3_-dopamine receptor availability in the nucleus caudatus. They extend these findings by revealing a similar association with D_2_/D_3_-dopamine receptor availability in the right IFC, a key region of the neural stopping network.

Our study has limitations: First, [^18^F]fallypride has a comparable affinity to D_2_ and D_3_ dopamine receptors in vivo. Therefore, we are not able to distinguish specific findings for each receptor subtype. Second, using this tracer we cannot report findings in the D_1-_ dopamine receptor system which also plays a crucial role for successful motor inhibition. Another limitation is the lack of a control group for the behavioral findings in our participants with the OPRM1 G-allele. However, a previous study that investigated response inhibition by means of the SST in alcohol dependent OPRM1 G and A allele carriers as control group found no group differences regarding the SSRT (Courtney et al., 2013). Finally, there were several days between the behavioral/fMRI and PET assessments. Therefore, we cannot rule out the occurrence of events that potentially alter the D_2_/D_3_ receptor status in between the two measures.

In summary, the present study partially replicates previous findings and provides further evidence that dopamine receptor availability in the dorsal striatum is important for successful stopping (Eagle and Robbins, 2003, Ghahremani et al., 2012; Robertson et al., 2015). In addition, our results extend previous findings and suggest that dopamine receptor availability in the right inferior frontal cortex, a key region of the stopping network, is also strongly associated with stopping-related striatal fMRI activity in healthy OPRM1 G-allele carriers. Since we performed correlational analyses interpretations about causal relations of dopamine receptor availability and stopping-related fMRI activity are beyond the scope of this study.

## Supplementary Information

Below is the link to the electronic supplementary material.

Supplementary file1 (DOCX 229 KB)

## Data Availability

Material has not been reproduced from prior publications, whether by the same or different authors. Any previously published material is explicitly quoted and referenced. Prior publications or other submissions on the same data set or problem have been disclosed.

## References

[CR1] Adams ZW, Roberts WM, Milich R, Fillmore MT (2011). Does response variability predict distractibility among adults with attention-deficit/hyperactivity disorder?. Psychological Assessment.

[CR2] Albrecht DA, Kareken DA, Christian BT, Dzemidzic M, Yoder KK (2014). Cortical dopamine release during a behavioral response inhibition task. Synapse.

[CR3] Aron AR (2011). From reactive to proactive and selective control: Developing a richer model for stopping inappropriate responses. Biological Psychiatry.

[CR4] Aron AR, Poldrack RA (2006). Cortical and subcortical contributions to Stop-signal response inhibition: Role of the subthalamic nucleus. Journal of Neuroscience.

[CR5] Aron AR, Robbins TR, Poldrack RA (2014). Inhibition and the right inferior frontal cortex: One decade on. Trends in Cognitive Sciences.

[CR6] Ashburner J (2007). A fast diffeomorphic image registration algorithm. NeuroImage.

[CR7] Bari A, Robbins TW (2013). Inhibition and impulsivity: Behavioral and neural basis of response control. Progress in Neurobiology.

[CR8] Bari A, Mar AC, Theobald DE, Elands SA, Oganya KC, Eagle DM, Robbins TW (2011). Prefrontal and monoaminergic contributions to stop-signal task performance in rats. Journal of Neuroscience.

[CR9] Boehler CN, Appelbaum LG, Krebs RM, Hopf JM, Woldorff MG (2010). Pinning down response inhibition in the brain–conjunction analyses of the Stop-signal task. NeuroImage.

[CR10] Bosker WM, Neuner I, Shah NJ (2017). The role of impulsivity in psychostimulant- and stress-induced dopamine release: Review of human imaging studies. Neuroscience & Biobehavioral Reviews.

[CR11] Buckholtz JW, Treadway MT, Cowan RL, Woodward ND, Li R, Ansari MS (2010). Dopaminergic network differences in human impulsivity. Science.

[CR12] Chamberlain SR, Sahakian BJ (2007). The neuropsychiatry of impulsivity. Current Opinion in Psychiatry.

[CR13] Chambers CD, Garavan H, Bellgrove MA (2009). Insights into the neural basis of response inhibition from cognitive and clinical neuroscience. Neuroscience & Biobehavioral Reviews.

[CR14] Chen W, Hemptinne C, Miller AM, Leibbrand M, Little SJ, Lim DA (2020). Prefrontal-subthalamic hyperdirect pathway modulates movement inhibition in humans. Neuron.

[CR15] Cieslik EC, Mueller VI, Eickhoff CR, Langner R, Eickhoff SB (2015). Three key regions for supervisory attentional control: Evidence from neuroimaging meta-analyses. Neuroscience & Biobehavioral Reviews.

[CR16] Colzato LS, van den Wildenberg WP, van Wouwe NC, Pannebakker MM, Hommel B (2009). Dopamine and inhibitory action control: Evidence from spontaneous eye-blink rates. Experimental Brain Research.

[CR17] Courtney KE, Ghahremani DG, Ray LA (2013). Fronto-striatal functional connectivity during response inhibition in alcohol dependence. Addiction Biology.

[CR18] Cremers HR, Wager TD, Yarkoni T (2017). The relation between statistical power and inference in fMRI. PLoS ONE.

[CR19] Cyders MA, Coskunpinar A (2011). Measurement of constructs using self-report and behavioral lab tasks: Is there overlap in nomothetic span and construct representation for impulsivity?. Clinical Psychology Review.

[CR20] Desikan RS, Segonne F, Fischl B, Quinn BT, Dickerson BC, Blacker D (2006). An automated labeling system for subdividing the human cerebral cortex on MRI scans into gyral based regions of interest. NeuroImage.

[CR21] de Wit H, Enggasser JL, Richards JB (2002). Acute administration of d-amphetamine decreases impulsivity in healthy volunteers. Neuropsychopharmacology.

[CR22] Eagle DM, Robbins TW (2003). Lesions of the medial prefrontal cortex or nucleus accumbens core do not impair inhibitory cortex in rats performing a stop-signal reaction time task. Behavioural Brain Research.

[CR23] Eagle DM, Wong JC, Allan ME, Mar AC, Theobald DE, Robbins TW (2011). Contrasting roles for dopamine D1 and D2 receptor subtypes in the dorsomedial striatum but not the nucleus accumbens core during behavioral inhibition in the stop-signal task in rats. Journal of Neuroscience.

[CR24] Eickhoff SB, Stephan KE, Mohlberg H, Grefkes C, Fink GR, Amunts K, Zilles K (2005). A new SPM toolbox for combining probabilistic cytoarchitectonic maps and functional imaging data. NeuroImage.

[CR25] Eickhoff SB, Paus T, Caspers S, Grosbras MH, Evans AC, Zilles K (2007). Assignment of functional activations to probabilistic cytoarchitectonic areas revisited. NeuroImage.

[CR26] Forbes EE, Brown SM, Kimak M, Ferrell RE, Manuck SB, Hariri AR (2009). Genetic variation in components of dopamine neurotransmission impacts ventral striatal reactivity associated with impulsivity. Molecular Psychiatry.

[CR27] Ghahremani DG, Lee B, Robertson CL, Tabibnia G, Morgan AT, De Shetler N (2012). Striatal dopamine D_2_/D_3_ receptors mediate response inhibition and related activity in frontostriatal neural circuitry in humans. Journal of Neuroscience.

[CR28] Heckemann RA, Hajnal JV, Aljabar P, Rueckert D, Hammers A (2006). Automatic anatomical brain MRI segmentation combining label propagation and decision fusion. NeuroImage.

[CR29] Kayser AS, Vega T, Weinstein D, Peters J, Mitchell JM (2016). Right inferior frontal cortex activity correlates with tolcapone responsivity in problem and pathological gamblers. NeuroImage: Clinical.

[CR30] Keuken MC, Bazin P, Crown L, Hootsmans J, Laufer A, Müller-Axt,  (2014). Quantifying inter-individual anatomical variability in the subcortex using 7 T structural MRI. NeuroImage.

[CR31] Kohno M, Okita K, Morales AM, Robertson CL, Dean AC, Ghahremani DG (2016). Midbrain functional connectivity and ventral striatal dopamine D2-type receptors: Link to impulsivity in methamphetamine users. Molecular Psychiatry.

[CR32] Koob GF, Volkow N (2010). Neurocircuitry of addiction. Neuropsychopharmacology.

[CR33] Lammertsma AA, Hume SP (1996). Simplified reference tissue model for PET receptor studies. NeuroImage.

[CR34] Landvogt C, Buchholz HG, Bernedo V, Schreckenberger M, Werhahn KJ (2010). Alteration of dopamine D2/D3 receptor binding in patients with juvenile myoclonic epilepsy. Epilepsia.

[CR35] Lee HY, Wu TF, Tsai JD, Yang EL (2016). Applicability of the stop-signal task for preschoolers with ADHD. Perceptual and Motor Skills.

[CR36] Lehrl S, Triebig G, Fischer B (1995). Multiple choice vocabulary test MWT as a valid and short test to estimate premorbid intelligence. Acta Neurologica Scandinavica.

[CR37] Logan GD, Cowan WB (1984). On the ability to inhibit thought and action: A theory of an act of control. Psychological Review.

[CR38] Lorenz RC, Gleich T, Buchert R, Schlagenhauf F, Kühn S, Gallinat J (2015). Interactions between glutamate, dopamine, and the neuronal signature of response inhibition in the human striatum. Human Brain Mapping.

[CR39] Morein-Zamir S, Robbins TW (2015). Fronto-striatal circuits in response-inhibition: Relevance to addiction. Brain Research.

[CR40] Munafò MR, Nosek BA, Bishop DVM, Button KS, Chambers CD, Percie du Sert N (2017). A manifesto for reproducible science. Nature Human Behaviour.

[CR41] Nandam LS, Hester R, Wagner J, Dean AJ, Messer C, Honeysett A (2013). Dopamine D_2_ receptor modulation of human response inhibition and error awareness. Journal of Cognitive Neuroscience.

[CR42] Olmstead MC, Ouagazzal AM, Kieffer BL (2009). Mu and delta opioid receptors oppositely regulate motor impulsivity in the signaled nose poke task. PLoS One.

[CR43] Overtoom CC, Kenemans JL, Verbaten MN, Kemner C, van der Molen M, van Engeland H (2002). Inhibition in children with attention-deficit/hyperactivity disorder: A psychophysiological study of the stop task. Biological Psychiatry.

[CR44] Perri RL, Spinelli D, Di Russo F (2017). Missing the target: The neural processing underlying the omission error. Brain Topography.

[CR45] Pfeifer P, Sariyar M, Eggermann T, Zerres K, Vernaleken I, Tüscher O (2015). Alcohol consumption in healthy OPRM1 G Allele carriers and its association with impulsive behavior. Alcohol and Alcoholism.

[CR46] Pfeifer P, Tüscher O, Buchholz HG, Gründer G, Vernaleken I, Paulzen M (2017). Acute effect of intravenously applied alcohol in the human striatal and extrastriatal D2 /D3 dopamine system. Addiction Biology.

[CR47] Ray LA, Hutchison KE (2012). A polymorphism of the mu-opioid receptor gene (OPRM1) and sensitivity to the effects of alcohol in humans. Alcoholism: Clinical and Experimental Research.

[CR48] Reynolds B, Ortengren A, Richards JB, de Wit H (2006). Dimensions of impulsive behavior: Personality and behavioral measures. Personality and Individual Differences.

[CR49] Robertson CL, Ishibashi K, Mandelkern MA, Brown AK, Ghahremani DG, Sabb F (2015). Striatal D1- and D2-type dopamine receptors are linked to motor response inhibition in human subjects. Journal of Neuroscience.

[CR50] Robbins TW, Gillan CM, Smith DG, de Wit S, Ersche KD (2012). Neurocognitive endophenotypes of impulsivity and compulsivity: Towards dimensional psychiatry. Trends in Cognitive Sciences.

[CR51] Sebastian A, Gerdes B, Feige B, Klöppel S, Lange T (2012). Neural correlates of interference inhibition, action withholding and action cancelation in adult ADHD. Psychiatry Research: Neuroimaging.

[CR52] Sebastian A, Pohl MF, Klöppel S, Feige B, Lange T, Stahl C (2013). Disentangling common and specific neural subprocesses of response inhibition. NeuroImage.

[CR53] Sebastian A, Jung P, Krause-Utz A, Lieb K, Schmahl C, Tüscher O (2014). Frontal dysfunctions of impulse control - a systematic review in borderline personality disorder and attention-deficit/hyperactivity disorder. Frontiers in Human Neuroscience.

[CR54] Sebastian, A., Jung, P., Neuhoff, J., Wibral, M., Fox, P. T., Lieb, K., et al. (2016). Dissociable attentional and inhibitory networks of dorsal and ventral areas of the right inferior frontal cortex: a combined task-specific and coordinate-based meta-analytic fMRI study. *Brain Structure and Function,**221*, 1635–1651. 10.1007/s00429-015-0994-y PMC4791198 25637472

[CR55] Sebastian, A., Rössler, K., Wibral, M., Mobascher, A., Lieb, K., Jung, P., et al. (2017). Neural architecture of selective stopping strategies: distinct brain activity patterns are associated with attentional capture but not with outright stopping. *Journal of Neuroscience,* *37*, 9785–9794. 10.1523/JNEUROSCI.1476-17.2017 PMC6596607 28887387

[CR56] Stahl C, Voss A, Schmitz F, Nuszbaum M, Tüscher O, Lieb K (2014). Behavioral components of impulsivity. Journal of Experimental Psychology: General.

[CR57] Turner D, Sebastian A, Tüscher O (2017). Impulsivity and cluster B personality disorders. Current Psychiatry Reports.

[CR58] Verbruggen F, Logan GD (2008). Response inhibition in the stop-signal paradigm. Trends in Cognitive Sciences.

[CR59] Verbruggen F, Aron AR, Band GP, Beste C, Bissett PG, Brockett AT (2019). A consensus guide to capturing the ability to inhibit actions and impulsive behaviors in the stop-signal task. eLife.

[CR60] Wiskerke J, Schetters D, van Es IE, van Mourik Y, den Hollander BR, Schoffelmeer AN (2011). μ-Opioid receptors in the nucleus accumbens shell region mediate the effects of amphetamine on inhibitory control but not impulsive choice. Journal of Neuroscience.

[CR61] Wittchen, H., & Pfister, H.U. (1997). DIA-X interview. Frankfurt.

