# Supplementary material for: Prefrontal and striatal dopamine D2/D3 receptors correlate with fMRI BOLD activation during stopping
Source: Brain Imaging Behav. 2021 Aug 17;16(1):186–98. doi: 10.1007/s11682-021-00491-y (PMC8825403; doi:10.1007/s11682-021-00491-y)
Supplement: Supplementary file 1 — Supplementary file1 (DOCX 229 KB) [file 11682_2021_491_MOESM1_ESM.docx]

**Supplemental material to:**

**Prefrontal and striatal dopamine D_2_/D_3_ receptors correlate with fMRI BOLD activation during stopping**

Philippe Pfeifer ^a#$^, Alexandra Sebastian ^b$, c^, Hans Georg Buchholz ^d^, Christoph P. Kaller ^e^, Gerhard Gründer ^f^, Christoph Fehr ^g^, Mathias Schreckenberger ^d^, Oliver Tüscher ^b, c^

^a^ University Hospital of Psychiatry Bern, Bern, Switzerland

^b^ Department of Psychiatry and Psychotherapy, University Medical Center of the Johannes Gutenberg University Mainz, Untere Zahlbacher Straße 8, 55131 Mainz, Germany

^c^ Leibniz Institute for Resilience Research, Wallstraße 7, 55122 Mainz, Germany

^d^ Department of Nuclear Medicine, University Medical Center of the Johannes Gutenberg University Mainz, Langenbeckstraße 1, 55131 Mainz, Germany

^e^ Department of Neurology and Neuroscience, University Medical Centre Freiburg, Germany; Freiburg Brain Imaging Centre, University Medical Centre Freiburg, Germany; Brain Links-BrainTools Cluster of Excellence, University Medical Centre Freiburg, Germany

^f^ Central Institute of Mental Health, Department of Molecular Neuroimaging, Medical Faculty Mannheim, University of Heidelberg, Mannheim, Germany

^g^ Department of Psychiatry, Psychotherapy and Psychosomatics, Agaplesion Markus Hospital, Wilhelm-Epstein-Straße 4, 60431 Frankfurt/Main, Germany

^#^ corresponding author

^$^ both authors contributed equally

**Address for correspondence:**

Philippe Pfeifer, MD.

Universitäre Psychiatrische Dienste Bern

Universitätsklinik für Psychiatrie und Psychotherapie

Bolligenstrasse 111

3000 Bern 60, Switzerland

Telephone: +41 (0)319309252

Mail: Philippe.Pfeifer@upd.ch


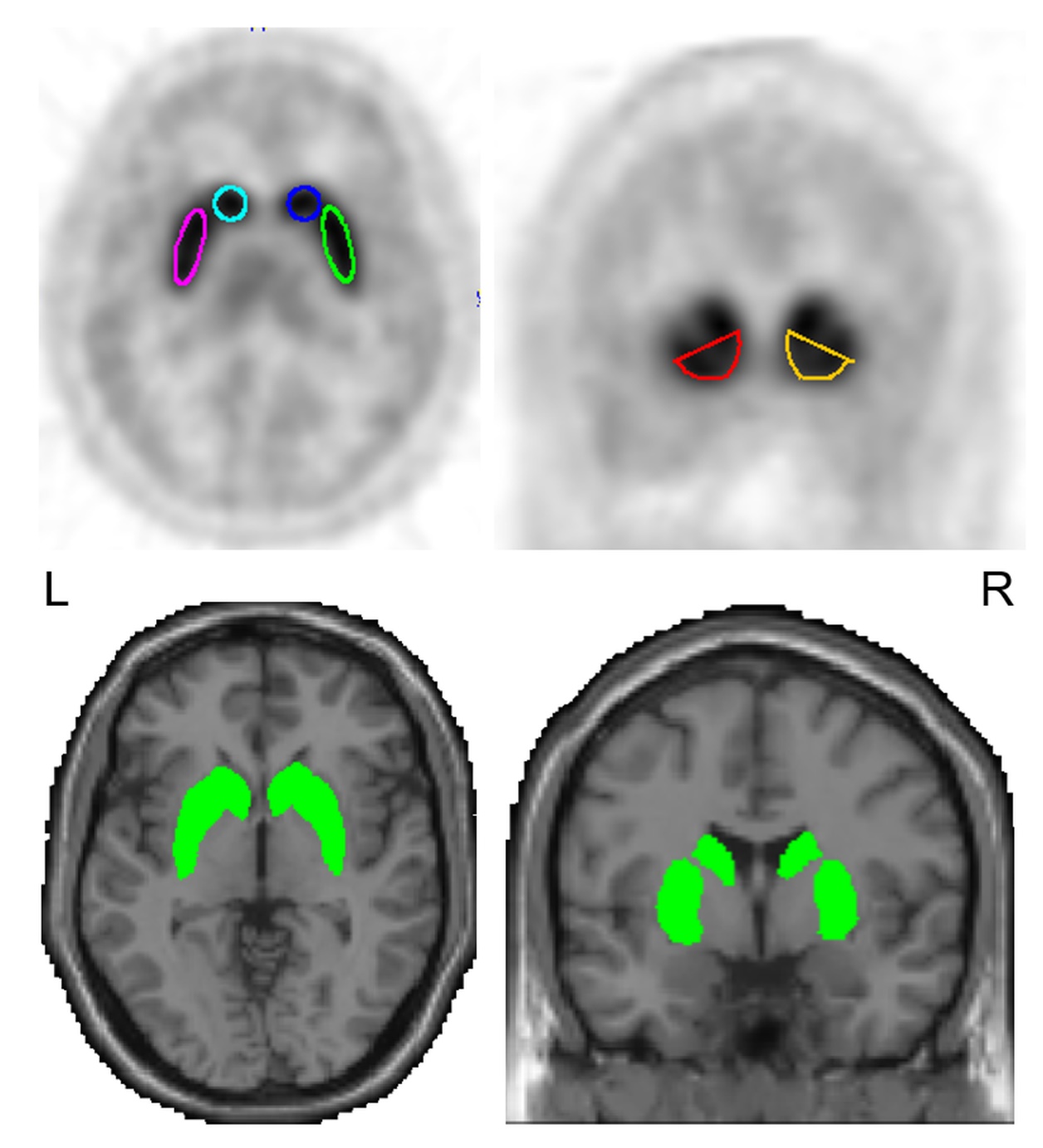


**Supplemental Figure S1**. Striatal masks used for PET data (top row) and for fMRI data (bottom row). Please note that for PET data separate masks for caudate and putamen were used, whereas for fMRI data a striatal mask combining caudate and putamen was used. For PET data, the BP_ND_ was calculated on Volumes-of-Interests (VOI) by applying a VOI-template that we used in a preceding publication (Landvogt et al., 2010). For fMRI data, we used the left and right striatum VOIs from the probabilistic atlas from Keuken et al. (2014) thresholded at 10%.

**References**

Keuken, M.C., Bazin, P., Crown, L., Hootsmans, J., Laufer, A., Müller-Axt, et al., 2014. Quantifying inter-individual anatomical variability in the subcortex using 7 T structural MRI. NeuroImage. 94:40–46.

Landvogt, C., Buchholz, H.G., Bernedo, V., Schreckenberger, M., Werhahn, K.J., 2010. Alteration of dopamine D2/D3 receptor binding in patients with juvenile myoclonic epilepsy. Epilepsia, 51, 1699-706.
